# Primary Stenting Immediatly after Surgery in Occluded Anastomoses of Aortoaortic Tube Graft: A Case Report

**DOI:** 10.4061/2010/521326

**Published:** 2010-07-20

**Authors:** M. Rabellino, L. García-Nielsen, T. Zander, S. Baldi, A. Estigarribia, I. Zerolo, H. Cheves, R. Llorens, M. Maynar

**Affiliations:** ^1^Department of Endovascular Therapy, Hospital Hospiten Rambla, General Franco 115, 38001 Santa Cruz de Tenerife, Spain; ^2^Department of Interventional Cardiology, Hospital Hospiten Rambla, General Franco 115, 38001 Santa Cruz de Tenerife, Spain; ^3^Department of Cardiovascular Surgery, Hospital Hospiten Rambla, General Franco 115, 38001 Santa Cruz de Tenerife, Spain

## Abstract

The conventional elective open procedures for abdominal aortic aneurysm repair are reliable and yield durable results. The aortoaortic tube graft has the lowest morbidity incidence when compared with different techniques. Albeit infrequent, thrombosis can be present in the first 30 days. Its treatment consists in thrombectomy and anastomosis evaluation, but with an increase in morbidity, especially in patients with urgent reintervention. This is a case report of a patient with aortoaortic tube graft, who present critical left limb ischemia immediately after surgical procedure. Angiography showed complete occlusion of left common iliac artery, affecting the distal graft anastomosis. The occlusion was resolved with endovascular treatment, and a noncovered, self-expanding, nitinol stent was deployed (primary stenting) covering the distal bypass anastomosis, with no complications and complete lower limb perfusion recovery. One month later, the patient was still asymptomatic, with distal pulse palpable and ankle-brachial index 1.

## 1. Introduction

Surgical treatment has been the most widespread therapy for infrarenal abdominal aneurysm. Different techniques, like aortobiiliac and aorto-bifemoral bifurcated graft, or aortoaortic tube graft are used. The latter has the lowest morbidity incidence [1]. 

Despite the low incidence, thrombosis can be present in the first days after surgical procedure, mainly due to anastomosis sites problems. Thrombectomy and anastomosis evaluation is indicated in this situation [2]. 

We report a case of a patient with an infrarenal aorta aneurysm, who was operated with an aortoaortic tube graft technique and started with acute left limb ischemia 1 hour immediately after the surgical procedure. Angiography showed total occlusion of the left common iliac artery, involving the distal anastomosis site of the graft. The occlusion was successfully resolved with endovascular procedure, and a noncovered, self-expanding, nitinol stent was deployed, covering the distal bypass anastomosis, with complete distal flow restoration.

## 2. Case Report

**Figure 1:**
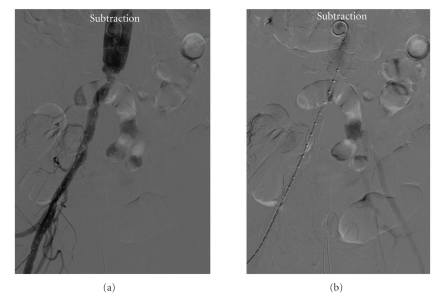
(a) Angiographic image showing the distal aortoaortic tube graft anastomosis. It also appreciates the left common iliac artery occlusion. (b) Angiographic image showing external iliac artery being perfused from the ipsilateral internal iliac artery.

**Figure 2:**
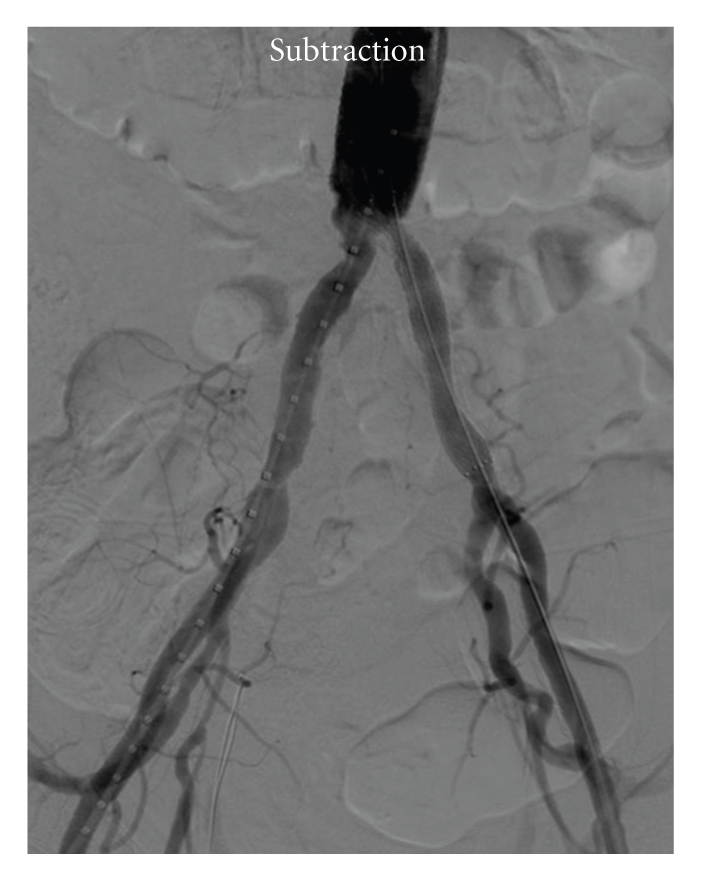
Final result after stent deployment and 6 mm balloon angioplasty. Left common iliac artery flow was restored without images of contrast extravasations.

A 73-year-old man, with hypertension, hyperlipemia, noninsulin dependent diabetes, and previous coronary percutaneous revascularization, was admitted to our hospital for surgical repair of a 60 mm diameter infrarenal abdominal aortic aneurysm. Surgical technique was aortoartic tube graft with a 22 mm diameter. Proximal anastomosis was at infrarenal aorta segment while distal anastomosis was at both primitive iliac arteries ostium. Finally, the patient was sent to intensive care unit to continue postoperative recovery. One hour late, the patient suddenly felt left limb pain at rest. Physical examination revealed pale skin, coldness, and distal pulse absence. He underwent angiography diagnostic procedure through 5 f sheath placed in the right femoral artery. Patency of the aortoaortic bypass was confirmed but left common iliac artery was found to be occluded from its ostium, in coincidence with the distal anastomosis of the graft (Figures 1(a) and 1(b)). Heavily calcified lesions were observed at fluoroscopy. External iliac artery received blood perfusion from hypogastric artery with patency of the femoropopliteal segment. A decision to endovascular recanalization was made, mainly to avoid a new open surgery intervention. A 6 fr sheath was placed into the left femoral artery in a retrograde way, and iliac artery was recanalized using a 0.035 hydrophilic glide wire (Terumo Medical Corp. Somerset, NJ.) and 5 Fr hydrophilic multipurpose guide catheter (Terumo Medical Corp. Somerset, NJ). Finally we decided to deliver a self-expanding, noncovered, nitinol stent, principally to avoid the rupture of the surgical suture with the pressure exerted by the balloon of a balloon-expandable stent in a less than 2 hours anastomosis. At the same time, covering the lesion with a primary stent instead other techniques, like thrombectomy, may reduce the possibility of distal embolization manly because embolic material is trapped between the stent and the arterial wall. A 9 × 60 mm Smart stent (Cordis, Miami Lakes, FL) was delivered covering the distal graft anastomosis, with flow restoration. A residual stenosis was observed, and a 6 × 40 mm Opta balloon (Cordis, Miami Lakes, FL) was inflated, but 30%–40% residual stenosis still persisted (Figure 2). We decided to end the procedure, principally because flow restoration was achieved, and the risk of suture rupture was high. 

**Figure 3:**
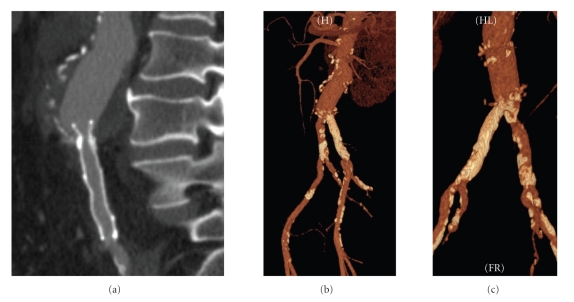
(a) Multidetector computed tomography (MDCT) image (multiplanar reconstruction) showing adequate stent expansion covering the distal graft anastomosis. (b) and (c) Tridimensional reconstruction (MDCT images, lateral and posterior views) showing the heave calcified lesions.

At the room, physical examination revealed normal skin color and temperature, with distal pulse palpable and ankle-brachial index (ABI) measure was 1. Scanner control images showed the stent covering the distal graft anastomosis and 30% residual stenosis (Figures 3(a) and 3(b)). 

The patient was sent home 6 days later with a double antiaggregation prescription with 100 mg/day aspirin and 75 mg/day clopidogrel during 3 months. At one-month control the patient remains asymptomatic.

## 3. Discusion

The conventional elective open procedures for abdominal aortic aneurysm repair are reliable and yield durable results [2]. Different techniques, like aorto-biiliac and aorto-bifemoral bifurcated graft or aortoaortic tube graft are performed. The latter has the lowest morbidity incidence [1], mainly due to the lowest time of procedure, has the less chance of damage to other organs veins, low incidence of sexual dysfunction, and less complications related to the anastomosis [1–4]. Thrombotic complications can be found in approximately 2% of cases, less commonly in the first 30 days [1]. Thrombectomy and anastomosis revision is the treatment of choice in these cases. It is important to remember that a luminal flap or technical defects are probably the most frequent causes of thrombosis in the first days, so inspection of the anastomosis is mandatory [5]. At the same time, morbimortality of re-interventions can reach 66% of nonelective procedures versus 3% of elective surgeries [6]. Currently, endovascular treatment of late complications related with graft anastomosis is well established as first-line therapy [7–10] while surgery is still the preferred treatment in case of acute complications. The main problem of endovascular treatment in patients with acute complications is the fear of burst open of suture. However, there is a report of experimental model in animals where they tested stents deployment in aorto-biiliac bypass during the surgery in order to evaluate intimal hyperplasia. The authors found no tears or suture dehiscence in the anastomosis [11]. In our case, we choose endovascular therapy, primarily to prevent the high morbidity in nonelective surgical re-intervention [6]. Moreover, the decision to use a self-expanding stent instead of a balloon expandable, a stent with less handling of release and liberation precision, was mostly based on the less capacity of damages of these stents. We have previously reported our experience with self-expandable stents in other territories [12]. The ability of the self-expanding stent to gradually grow in volume may allow deployment at lower pressures. Other potential benefit of this stent is its capacity to expand continually without the need for further angioplasty, reducing, in this case, the risk of suture dehiscence. However, the ability to gradually grow in volume may not be present in heavily calcified lesions. Fluoroscopy showed severe calcified lesions in left common iliac artery and may be the cause of the final 30%–40% residual stenosis in our patient. Nevertheless, skin color and distal pulse recovery were observed and the procedure ended, with the possibility of a new angioplasty in case of need in the followup. 

To our knowledge, this is the first report of an endovascular treatment in a case of acute aortoaortic tube graft thrombosis. Since we have found no previous report of self-expanding stent deployment in a <2 h aortoaortic tube graft anastomosis thrombosis, it is too early to draw firm conclusions. There is a need for a larger patient cohort to investigate feasibility and safety with a long-term clinical outcome.
